# Diaqua­dichloridobis[quinazolin-4(1*H*)-one-κ*N* ^3^]copper(II)

**DOI:** 10.1107/S1600536810048890

**Published:** 2010-11-27

**Authors:** Kambarali Turgunov, Shirin Shomurotova, Nasir Mukhamedov, Bakhodir Tashkhodjaev

**Affiliations:** a S.Yunusov Institute of the Chemistry of Plant Substances, Academy of Sciences of Uzbekistan, Mirzo Ulugbek Str. 77, Tashkent 100170, Uzbekistan; b Tashkent State Pedagogical University Named After Nizami, Yusuf Khos Khojib Str. 103, Tashkent 100100, Uzbekistan

## Abstract

In the title complex, [CuCl_2_(C_8_H_6_N_2_O)_2_(H_2_O)_2_], the Cu^II^ ion is located on an inversion center and is octahedrally coordinated by two N atoms of the 1*H*-quinazolin-4-one ligand, two chloride ligands and two aqua ligands. The axial Cu—O distances are significantly longer [2.512 (2) Å], than the Cu—N [2.022 (2) Å] and Cu—Cl [2.3232 (4) Å] distances as a result of Jahn–Teller distortion. Aqua ligands are involved in intra- and inter­molecular hydrogen bonding, and N—H⋯O inter­molecular hydrogen bonds are formed between the organic ligands. In addition, weak π–π inter­actions are observed between the benzene rings of the ligand [centroid–centroid distance = 3.678 (1) Å].

## Related literature

The crystal structure of pyrimidin-4(3*H*)-one was reported by Vaillancourt *et al.* (1998 ▶). For a Cd(II) coordination polymer with quinazolin-4(3*H*)-one, see: Turgunov & Englert (2010 ▶). For computational studies of quinazolin-4-one derivatives, see: Bakalova *et al.* (2004 ▶).
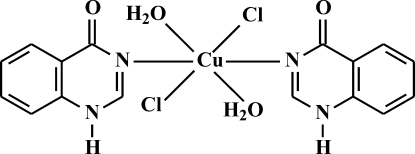


## Experimental

### 

#### Crystal data

[CuCl_2_(C_8_H_6_N_2_O)_2_(H_2_O)_2_]
*M* *_r_* = 462.77
Monoclinic, 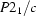

*a* = 6.7438 (3) Å
*b* = 18.5328 (8) Å
*c* = 6.7831 (3) Å
β = 90.735 (3)°
*V* = 847.69 (6) Å^3^
*Z* = 2
Cu *K*α radiation
μ = 5.03 mm^−1^
*T* = 293 K
0.55 × 0.35 × 0.20 mm

#### Data collection

Oxford Diffraction Xcalibur Ruby diffractometer
Absorption correction: multi-scan (*CrysAlis PRO*; Oxford Diffraction, 2007 ▶) *T*_min_ = 0.366, *T*_max_ = 1.000
5548 measured reflections
1725 independent reflections
1639 reflections with *I* > 2σ(*I*)
*R*_int_ = 0.040

#### Refinement

*R*[*F* ^2^ > 2σ(*F* ^2^)] = 0.032
*wR*(*F* ^2^) = 0.089
*S* = 1.10
1725 reflections
137 parameters
3 restraints
H atoms treated by a mixture of independent and constrained refinement
Δρ_max_ = 0.37 e Å^−3^
Δρ_min_ = −0.46 e Å^−3^

### 

Data collection: *CrysAlis PRO* (Oxford Diffraction, 2007 ▶); cell refinement: *CrysAlis PRO*; data reduction: *CrysAlis PRO*; program(s) used to solve structure: *SHELXS97* (Sheldrick, 2008 ▶); program(s) used to refine structure: *SHELXL97* (Sheldrick, 2008 ▶); molecular graphics: *XP* (Bruker, 1998 ▶); software used to prepare material for publication: *publCIF* (Westrip, 2010 ▶).

## Supplementary Material

Crystal structure: contains datablocks I, global. DOI: 10.1107/S1600536810048890/nk2075sup1.cif

Structure factors: contains datablocks I. DOI: 10.1107/S1600536810048890/nk2075Isup2.hkl

Additional supplementary materials:  crystallographic information; 3D view; checkCIF report

## Figures and Tables

**Table 1:** Hydrogen-bond geometry (Å, °)

*D*—H⋯*A*	*D*—H	H⋯*A*	*D*⋯*A*	*D*—H⋯*A*
O1*W*—H1*W*⋯O1^i^	0.84 (2)	1.92 (3)	2.732 (2)	162 (3)
O1*W*—H2*W*⋯Cl1^ii^	0.85 (2)	2.51 (2)	3.355 (2)	171 (4)
N1—H1⋯O1^iii^	0.84 (2)	2.39 (3)	3.022 (2)	133 (3)
N1—H1⋯Cl1^iv^	0.84 (2)	2.63 (3)	3.324 (2)	140 (3)
C2—H2*A*⋯O1*W*	0.93	2.38	2.972 (3)	121
C7—H7*A*⋯O1*W*^v^	0.93	2.57	3.421 (3)	152

Symmetry codes: (i) 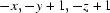; (ii) 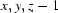; (iii) 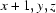; (iv) 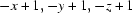; (v) 
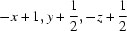
.

**Fig. 1.**
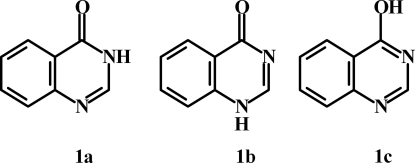
The 3H, 1H and 4-OH tautomers of 4-quinazolinone.

**Fig. 2.**
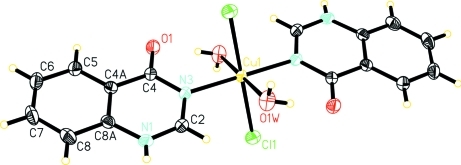
The molecular structure of the title complex with the atom-numbering scheme. Displacement ellipsoids are drawn at the 50% probability level.

**Fig. 3.**
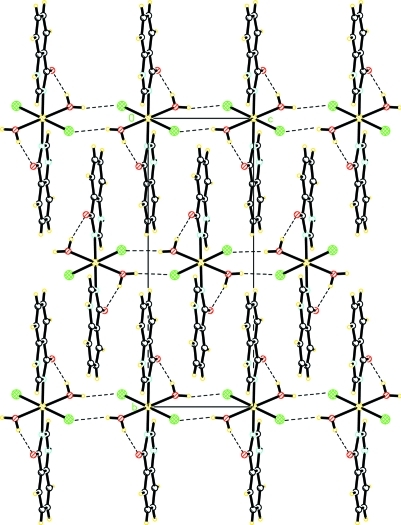
Crystal packing of the title compound viewed along the a axis, showing the formation of a hydrogen-bonded chain along [001]. Molecular complexes are furhter linked by π–π stacking interactions, formed between ligands, along [010] and [001] directions [Cg1···Cg1vi=3.678 (1) Å].

**Fig. 4.**
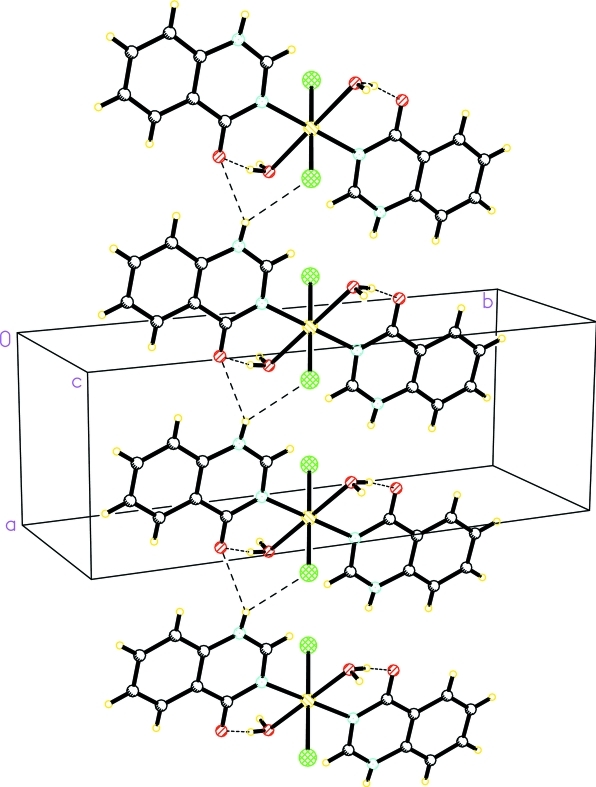
Part of the crystal structure of the title compound showing the formation of a hydrogen-bonded chain along [100].

## supplementary crystallographic information

### Comment

In solutions, 4-quinazolinone could have in principle three isomers—1*H*, 3*H*, and 4-O*H*, as shown in Figure 1, with preference of 3*H*-tautomer. Recently, the crystal structure of a Cd^II^ coordination complex has been reported, in which 3*H*-quinazolin-4-one (3*H*-tautomer) acted as a ligand (Turgunov & Englert, 2010). We now report the structure of a Cu^II^ complex in which 1*H*-quinazolin-4-one (1*H*-tautomer) acts as a ligand.

In the title compound, Cu^II^ ion is located on the inversion center and has an octahedral coordination environment: two ligands coordinated *via* N atoms in position 3, two chloride ligands and two aqua ligands (Figure 2). The distances between Cu and coordination atoms are the following: d(Cu—N3) = 2.022 (2) Å, d(Cu—Cl) = 2.3232 (4) Å and d(Cu—Ow) = 2.512 (2) Å. Long distances of metal-aqua bonds than other four coordination bonds indicate existence of the Jahn-Teller elongation effect.

Aqua ligands are involved in intramolecular and intermolecular hydrogen bonding. Intramoleculer H-bonding is occurring with carbonyl group of the ligand. An intermolecular H-bonding of aqua and chloride ligands gives raise to chains along [001] (Figure 3). In addition, between ligand and water molecules are formed weak C–H···O hydrogen bonds. Intermolecular N—H···O and N—H···Cl hydrogen bonds formed between the organic and chloride ligands link molecular complexes into hydrogen-bonded chains along [100] (Figure 4; Table 1). Weak π···π ring interactions connect the molecular complexes along [010] and [001] directions. [*Cg*1···*Cg*1^vi^=3.678 (1) Å, where *Cg*1=C4A–C5–C6–C7–C8–C8A; ^vi^ = *x*, 3/2 - *y*, 1/2 + *z*].

### Experimental

A solution of 17.05 mg (0.1 mmol) of copper(II) chloride dihydrate in 2 ml of water was added to a solution of 29.23 mg (0.2 mmol) of 3*H*-quinazolin-4-one in 5 ml of ethanol. The solution allowed to stand at room temperature for one week, after which light-blue crystals were obtained.

### Refinement

C-bound H atoms were positioned geometrically and treated as riding on their C atoms, with C—H distances of 0.93 Å (aromatic) and were refined with *U*_iso_(H)=1.2Ueq(C). N-bound H atoms and water H atoms involved in the intermolecular hydrogen bonding were found by difference Fourier synthesis and refined isotropically with a distance restrains of 0.87 (2) and 0.85 (2) Å, respectively [N—H =0.84 (2) Å, O1w—H1w=0.84 (2) Å, O1w—H2w=0.85 (2) Å].

### Crystal data

**Table:**

[CuCl_2_(C_8_H_6_N_2_O)_2_(H_2_O)_2_]	*F*(000) = 470
*M**_r_* = 462.77	*D*_x_ = 1.813 Mg m^−^^3^
Monoclinic, *P*2_1_/*c*	Cu *K*α radiation, λ = 1.54180 Å
Hall symbol: -P 2ybc	Cell parameters from 4533 reflections
*a* = 6.7438 (3) Å	θ = 4.8–75.3°
*b* = 18.5328 (8) Å	µ = 5.03 mm^−^^1^
*c* = 6.7831 (3) Å	*T* = 293 K
β = 90.735 (3)°	Prism, light-blue
*V* = 847.69 (6) Å^3^	0.55 × 0.35 × 0.20 mm
*Z* = 2	

### Data collection

**Table:**

Oxford Diffraction Xcalibur Ruby diffractometer	1725 independent reflections
Radiation source: Enhance (Cu) X-ray Source	1639 reflections with *I* > 2σ(*I*)
graphite	*R*_int_ = 0.040
Detector resolution: 10.2576 pixels mm^-1^	θ_max_ = 77.1°, θ_min_ = 4.8°
ω scans	*h* = −5→8
Absorption correction: multi-scan (*CrysAlis PRO*; Oxford Diffraction, 2007)	*k* = −23→22
*T*_min_ = 0.366, *T*_max_ = 1.000	*l* = −8→8
5548 measured reflections	

### Refinement

**Table:**

Refinement on *F*^2^	Secondary atom site location: difference Fourier map
Least-squares matrix: full	Hydrogen site location: inferred from neighbouring sites
*R*[*F*^2^ > 2σ(*F*^2^)] = 0.032	H atoms treated by a mixture of independent and constrained refinement
*wR*(*F*^2^) = 0.089	*w* = 1/[σ^2^(*F*_o_^2^) + (0.0535*P*)^2^ + 0.3464*P*] where *P* = (*F*_o_^2^ + 2*F*_c_^2^)/3
*S* = 1.10	(Δ/σ)_max_ < 0.001
1725 reflections	Δρ_max_ = 0.37 e Å^−^^3^
137 parameters	Δρ_min_ = −0.46 e Å^−^^3^
3 restraints	Extinction correction: *SHELXL97* (Sheldrick, 2008), Fc^*^=kFc[1+0.001xFc^2^λ^3^/sin(2θ)]^-1/4^
Primary atom site location: structure-invariant direct methods	Extinction coefficient: 0.0067 (7)

### Special details

**Table:**

Geometry. All e.s.d.'s (except the e.s.d. in the dihedral angle between two l.s. planes) are estimated using the full covariance matrix. The cell e.s.d.'s are taken into account individually in the estimation of e.s.d.'s in distances, angles and torsion angles; correlations between e.s.d.'s in cell parameters are only used when they are defined by crystal symmetry. An approximate (isotropic) treatment of cell e.s.d.'s is used for estimating e.s.d.'s involving l.s. planes.
Refinement. Refinement of *F*^2^ against ALL reflections. The weighted *R*-factor *wR* and goodness of fit *S* are based on *F*^2^, conventional *R*-factors *R* are based on *F*, with *F* set to zero for negative *F*^2^. The threshold expression of *F*^2^ > σ(*F*^2^) is used only for calculating *R*-factors(gt) *etc*. and is not relevant to the choice of reflections for refinement. *R*-factors based on *F*^2^ are statistically about twice as large as those based on *F*, and *R*- factors based on ALL data will be even larger.

### Fractional atomic coordinates and isotropic or equivalent isotropic displacement parameters (Å^2^)

**Table:**

	*x*	*y*	*z*	*U*_iso_*/*U*_eq_	
Cu1	0.0000	0.5000	0.5000	0.02135 (17)	
Cl1	0.20934 (7)	0.45671 (2)	0.74764 (6)	0.02750 (17)	
O1	−0.12427 (19)	0.66628 (7)	0.5870 (2)	0.0280 (3)	
N1	0.4508 (2)	0.65113 (9)	0.4461 (2)	0.0227 (3)	
C2	0.3358 (3)	0.59405 (10)	0.4636 (3)	0.0229 (4)	
H2A	0.3936	0.5490	0.4458	0.028*	
N3	0.1427 (2)	0.59595 (8)	0.5049 (2)	0.0209 (3)	
C4	0.0522 (3)	0.66252 (9)	0.5382 (3)	0.0198 (4)	
C4A	0.1738 (3)	0.72721 (10)	0.5123 (2)	0.0196 (4)	
C5	0.0922 (3)	0.79617 (10)	0.5345 (3)	0.0239 (4)	
H5A	−0.0399	0.8014	0.5693	0.029*	
C6	0.2088 (3)	0.85635 (11)	0.5046 (3)	0.0288 (4)	
H6A	0.1540	0.9022	0.5167	0.035*	
C7	0.4101 (3)	0.84875 (11)	0.4560 (3)	0.0311 (4)	
H7A	0.4871	0.8897	0.4363	0.037*	
C8	0.4945 (3)	0.78128 (11)	0.4373 (3)	0.0276 (4)	
H8A	0.6278	0.7764	0.4064	0.033*	
C8A	0.3762 (3)	0.72057 (10)	0.4656 (2)	0.0205 (4)	
O1W	0.2398 (3)	0.46004 (9)	0.2416 (2)	0.0356 (4)	
H1W	0.218 (5)	0.4172 (11)	0.274 (5)	0.057 (10)*	
H1	0.570 (3)	0.6432 (17)	0.417 (5)	0.054 (9)*	
H2W	0.219 (6)	0.462 (2)	0.118 (3)	0.065 (10)*	

### Atomic displacement parameters (Å^2^)

**Table:**

	*U*^11^	*U*^22^	*U*^33^	*U*^12^	*U*^13^	*U*^23^
Cu1	0.0191 (2)	0.0140 (2)	0.0308 (3)	−0.00062 (12)	−0.00334 (16)	0.00158 (13)
Cl1	0.0262 (3)	0.0276 (3)	0.0286 (3)	0.00254 (16)	−0.00262 (18)	0.00244 (16)
O1	0.0175 (6)	0.0225 (7)	0.0441 (8)	0.0005 (5)	0.0050 (5)	−0.0008 (6)
N1	0.0149 (7)	0.0243 (8)	0.0290 (8)	0.0010 (6)	0.0006 (6)	0.0016 (6)
C2	0.0209 (8)	0.0190 (8)	0.0289 (9)	0.0025 (7)	−0.0013 (7)	0.0001 (7)
N3	0.0189 (7)	0.0161 (7)	0.0278 (7)	0.0015 (5)	−0.0009 (6)	0.0013 (6)
C4	0.0198 (8)	0.0170 (8)	0.0224 (8)	−0.0001 (6)	−0.0017 (6)	−0.0003 (6)
C4A	0.0196 (8)	0.0200 (8)	0.0191 (7)	−0.0005 (6)	−0.0020 (6)	−0.0002 (6)
C5	0.0256 (9)	0.0208 (9)	0.0253 (9)	0.0006 (7)	−0.0017 (7)	−0.0013 (7)
C6	0.0395 (11)	0.0180 (9)	0.0287 (9)	−0.0010 (8)	−0.0041 (8)	−0.0005 (7)
C7	0.0387 (11)	0.0230 (10)	0.0315 (10)	−0.0141 (8)	−0.0021 (8)	0.0013 (7)
C8	0.0242 (9)	0.0306 (10)	0.0280 (9)	−0.0088 (8)	−0.0003 (7)	0.0016 (8)
C8A	0.0205 (8)	0.0218 (9)	0.0192 (7)	−0.0024 (7)	−0.0027 (6)	0.0006 (6)
O1W	0.0416 (9)	0.0273 (8)	0.0380 (9)	0.0003 (6)	0.0032 (7)	−0.0016 (6)

### Geometric parameters (Å, °)

**Table:**

Cu1—N3	2.0221 (15)	C4A—C5	1.400 (3)
Cu1—N3^i^	2.0221 (15)	C4A—C8A	1.410 (3)
Cu1—Cl1	2.3232 (4)	C5—C6	1.381 (3)
Cu1—Cl1^i^	2.3232 (4)	C5—H5A	0.9300
O1—C4	1.241 (2)	C6—C7	1.409 (3)
N1—C2	1.318 (2)	C6—H6A	0.9300
N1—C8A	1.389 (2)	C7—C8	1.380 (3)
N1—H1	0.841 (18)	C7—H7A	0.9300
C2—N3	1.336 (2)	C8—C8A	1.394 (3)
C2—H2A	0.9300	C8—H8A	0.9300
N3—C4	1.396 (2)	O1W—H1W	0.837 (18)
C4—C4A	1.464 (2)	O1W—H2W	0.848 (19)
			
N3—Cu1—N3^i^	180.0	C5—C4A—C4	120.84 (16)
N3—Cu1—Cl1	90.40 (4)	C8A—C4A—C4	120.04 (16)
N3^i^—Cu1—Cl1	89.60 (4)	C6—C5—C4A	119.75 (18)
N3—Cu1—Cl1^i^	89.60 (4)	C6—C5—H5A	120.1
N3^i^—Cu1—Cl1^i^	90.40 (4)	C4A—C5—H5A	120.1
Cl1—Cu1—Cl1^i^	180.0	C5—C6—C7	120.38 (19)
C2—N1—C8A	121.43 (15)	C5—C6—H6A	119.8
C2—N1—H1	116 (2)	C7—C6—H6A	119.8
C8A—N1—H1	122 (2)	C8—C7—C6	120.79 (17)
N1—C2—N3	125.02 (16)	C8—C7—H7A	119.6
N1—C2—H2A	117.5	C6—C7—H7A	119.6
N3—C2—H2A	117.5	C7—C8—C8A	118.77 (18)
C2—N3—C4	119.11 (15)	C7—C8—H8A	120.6
C2—N3—Cu1	116.00 (12)	C8A—C8—H8A	120.6
C4—N3—Cu1	124.80 (12)	N1—C8A—C8	121.77 (17)
O1—C4—N3	121.02 (16)	N1—C8A—C4A	117.05 (15)
O1—C4—C4A	121.77 (16)	C8—C8A—C4A	121.17 (17)
N3—C4—C4A	117.21 (15)	H1W—O1W—H2W	106 (3)
C5—C4A—C8A	119.11 (16)		

Symmetry codes: (i) −*x*, −*y*+1, −*z*+1.

### Hydrogen-bond geometry (Å, °)

**Table:**

*D*—H···*A*	*D*—H	H···*A*	*D*···*A*	*D*—H···*A*
O1W—H1W···O1^i^	0.84 (2)	1.92 (3)	2.732 (2)	162 (3)
O1W—H2W···Cl1^ii^	0.85 (2)	2.51 (2)	3.355 (2)	171 (4)
N1—H1···O1^iii^	0.84 (2)	2.39 (3)	3.022 (2)	133 (3)
N1—H1···Cl1^iv^	0.84 (2)	2.63 (3)	3.324 (2)	140 (3)
C2—H2A···O1W	0.93	2.38	2.972 (3)	121
C7—H7A···O1W^v^	0.93	2.57	3.421 (3)	152

Symmetry codes: (i) −*x*, −*y*+1, −*z*+1; (ii) *x*, *y*, *z*−1; (iii) *x*+1, *y*, *z*; (iv) −*x*+1, −*y*+1, −*z*+1;  (v) −*x*+1, *y*+1/2, −*z*+1/2.
